# Supplementary material for: The fungal peptide toxin Candidalysin activates the NLRP3 inflammasome and causes cytolysis in mononuclear phagocytes
Source: Nat Commun. 2018 Oct 15;9:4260. doi: 10.1038/s41467-018-06607-1 (PMC6189146; doi:10.1038/s41467-018-06607-1)
Supplement: Supplementary file 1 — Supplementary Information [file 41467_2018_6607_MOESM1_ESM.pdf]

Supplementary Information

The fungal peptide toxin Candidalysin activates the NLRP3 inflammasome and causes cytolysis in mononuclear phagocytes

Lydia Kasper, Annika König, Paul-Albert Koenig, Mark S. Gresnigt, Johannes Westman, Rebecca A. Drummond, Michail S. Lionakis, Olaf Groß, Jürgen Ruland, Julian R Naglik, and Bernhard Hube[*]

[*]Corresponding author: Bernhard Hube
E-mail: bernhard.hube@leibniz-hki.de

a related to figure 3b

human MDMs, IL-1β

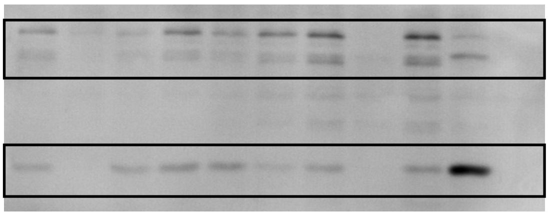

b related to figure 4c

murine BMDMs, IL-1β

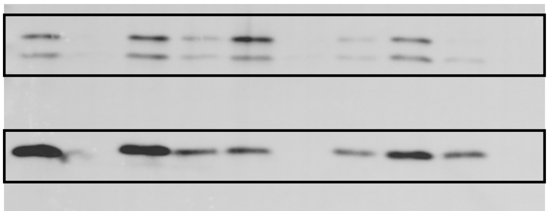

c related to figure 5e

human MDMs, Caspase-1

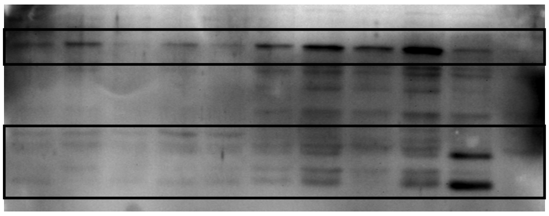

murine BMDMs, Caspase-1

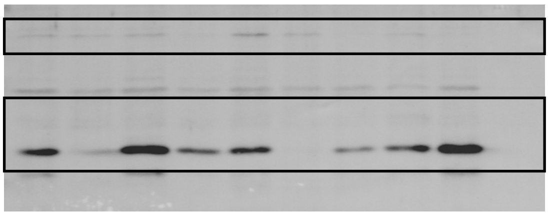

murine BMDCs, Caspase-1

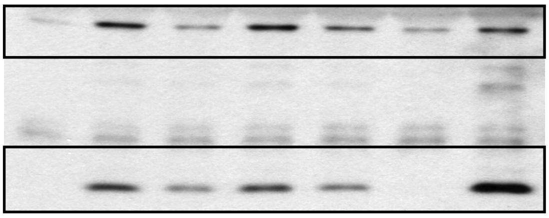

murine BMDCs, Caspase-1

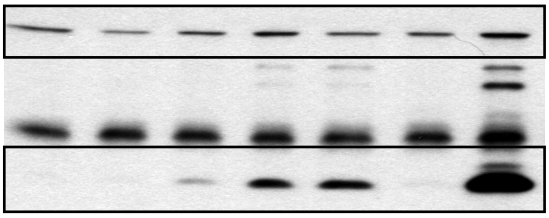

Supplementary Figure 1: Uncropped images of western blots shown in Fig. 3b (a), Fig. 4c (b) and Fig. 5e (c).
